# Findings and feasibility of major salivary gland ultrasound in childhood-onset systemic lupus erythematosus: a pilot study

**DOI:** 10.1186/s12969-021-00561-x

**Published:** 2021-05-17

**Authors:** Joseph McDonald, Patricia Vega-Fernandez, Tracy Ting

**Affiliations:** Division of Pediatric Rheumatology, Cincinnati Children’s Hospital Medical Center, Cincinnati, OH USA

**Keywords:** Ultrasound, Systemic lupus erythematosus, Sjögren’s syndrome, Salivary gland

## Abstract

**Background:**

Childhood-onset systemic lupus erythematosus (cSLE) is a complex autoimmune disorder with multi-organ manifestations and can be associated with other rheumatic diseases including Sjögren’s syndrome (SS). Salivary gland ultrasound (SGUS) represents a noninvasive tool to screen for salivary gland disease in rheumatic disease patients. The aims of this cross-sectional study were to determine feasibility of major SGUS in a clinic setting and to identify characteristics in a cohort of cSLE patients (without confirmed SS) that may be associated with salivary gland abnormalities consistent with secondary SS.

**Methods:**

Patients with SLE onset prior to age 18 were recruited. Patients completed questionnaires rating symptoms and underwent major SGUS examination. Disease and demographic differences were compared between cSLE patients with abnormal SGUS vs. cSLE patients with normal SGUS using t-tests and Fisher’s exact tests.

**Results:**

Thirty-one cSLE patients were recruited, 84% were female, 55% were Caucasian. The average disease duration among all patients was 5 years. Average time to complete the SGUS examination and scoring protocol was 7 min. 35% of SGUS scores were abnormal and significantly associated with IgG level at diagnosis, and anti-Ro and anti-La antibodies.

**Conclusions:**

This is one of the first studies to our knowledge that assesses major SGUS in a cohort of patients with cSLE without prior diagnoses of SS. The SGUS protocol was feasible to perform by rheumatologists in a clinic setting. Although the sample size was small, SGUS abnormalities were identified in one-third of patients. IgG level at diagnosis and anti-Ro and anti-La antibodies may be associated with SGUS abnormalities.

## Background

Systemic Lupus Erythematosus (SLE) is a complex autoimmune disease characterized by the presence of various autoantibodies and clinical manifestations affecting multiple organ systems. Childhood-onset Systemic Lupus Erythematosus (cSLE) is estimated to account for 10–20% of all cases of SLE [1]. Patients with SLE have an increased risk for the development of other autoimmune diseases. One disease that is clinically related and may coexist with SLE is Sjögren’s syndrome (SS). SS is an autoimmune disease that primarily causes inflammation and dysfunction of exocrine glands leading to the hallmark symptoms of dry eyes and dry mouth but can also cause other systemic manifestations and can lead to lymphoma [2]. Furthermore, additional long-term complications of SS include oral and ocular damage.

SS can occur on its own as a primary disease process (primary SS) or can occur in the setting of an additional rheumatic disease including SLE (known as secondary SS). Adult studies suggest a prevalence of secondary SS ranging from 14 to 18% in patients with SLE [3, 4]. The prevalence of secondary SS in cSLE is unclear and reports are isolated to smaller case studies [5–7].

Diagnoses of both primary and secondary SS in children is rare, however, its true prevalence is likely underestimated given current limitations in diagnostic criteria particularly when applied to pediatric patients [8]. Diagnostic criteria focus on dry mouth and dry eye symptoms that are not as commonly reported in children. Additionally, diagnosis is often confirmed by objective evaluation of salivary gland involvement and has traditionally been performed using parotid sialography and/or salivary scintigraphy, however these tests are invasive, require exposure to radiation, and are rarely used in pediatric practice [9].

Hence, there is a great need for a safe and quick tool to identify salivary gland abnormalities. Ultrasound provides a non-invasive, non-ionizing imaging technique that can be performed readily at the bedside and is particularly well-suited to pediatric practice. Ultrasound was first shown as a potentially useful tool for assessing salivary gland abnormalities in adult patients with primary and secondary SS as early as 1992 [10]. Major salivary glands normally have a homogenous appearance that is typically hyperechoic compared to surrounding facial muscles. In SS the major salivary glands have diffuse heterogenous appearance with numerous hypoechoic foci [11]. Several studies that have been conducted in patients with SS have shown correlations between these abnormalities identified on ultrasound and autoantibodies and clinical symptoms [12, 13].

Thus, ultrasound may play a role in identifying salivary gland abnormalities in cSLE patients who may be at risk for the development of secondary SS. This may lead to earlier diagnosis in children without overt symptoms of dry mouth or dry eyes and allow for targeted therapeutic interventions to prevent long-term damage.

One recent multicenter study that focused on juvenile SS and found correlations between salivary gland ultrasound abnormalities and dry mouth and eye symptoms, hyposalivation, and autoantibody status [14]. However, no studies have focused on cSLE patients who do not yet have a diagnosis of secondary SS. The aims of this study were to 1) determine feasibility of major salivary gland ultrasound (SGUS) in a pediatric rheumatology clinic setting and 2) identify disease characteristics in a cohort of cSLE patients (without confirmed SS) that may be associated with salivary gland abnormalities consistent with secondary SS.

## Methods

### Study group

The cross-sectional study enrolled 31 patients followed at Cincinnati Children’s Hospital Medical Center pediatric rheumatology department. All subjects over the age of 18 years provided informed consent. Those participants under age 18 years provided assent and parental consent. The Institutional Review Board of Cincinnati Children’s Hospital Medical Center approved the study.

Inclusion criteria included those patients with a diagnosis of childhood-onset systemic lupus erythematosus (cSLE). These patients were classified as cSLE if they had received a SLE diagnosis prior to the age of 18. Patient SLE diagnosis was based on American College of Rheumatology criteria per their primary pediatric rheumatologist. Exclusion criteria were any patient with a known salivary gland disorder (ex. sialolithiasis) or any patient with another primary rheumatic disease including primary SS and Mixed Connective Tissue Disease (MCTD).

Demographic characteristics, disease duration, medication use, and disease activity at the time of the visit were collected. Disease activity was measured using the Systemic Lupus Erythematosus Disease Activity Index (SLEDAI), a validated disease activity index in both adults and children [15]. SLEDAI scores of 1–5 are representative of mild disease activity, scores of 6–10 are moderate disease activity, scores of 11–19 are high disease activity, and scores > 20 are very high disease activity [15]. Specific laboratory investigations were also examined. Lab characteristics of interest included antibodies specific to SLE and/or SS: anti ds-DNA, anti-Ro, anti-La, anti-Sm, and anti-RNP, in addition to rheumatoid factor (RF), and IgG level at disease diagnosis. A questionnaire adapted from the European League Against Rheumatism (EULAR) Sjögren’s Syndrome Patient Reported Index (ESSPRI) with five questions was administered to all patients to assess their symptoms of dryness, fatigue, and pain [16]. They were asked to rate these symptoms from 0 to 10 over the past two weeks. The questionnaire included two additional questions. The first question asked the patient if they had any history of needing water to help swallow food. This was included because some pediatric patients have difficulty describing dryness symptoms. The second question asked if there was any history of recurrent glandular swelling since parotitis is a commonly reported symptom in children with SS [17, 18].

### US of salivary glands (SGUS)

Each patient underwent a one-time major SGUS using a real-time, high-resolution ultrasound machine (General Electric Logiq S8) with an 8–15 MHz linear transducer. Ultrasound examination included static B-mode images. A still image of each patient’s thyroid gland was obtained as a control for comparison, representing the expected homogenous appearance of a major salivary gland [11]. Images of the bilateral parotid glands were obtained in two orthogonal planes and images of the bilateral submandibular glands were obtained in one plane, as per previous acquisition protocol [12]. A longitudinal image of the parotid gland was obtained with the probe parallel to the ramus of the mandible just anterior to the ear. A transverse image of the parotid gland was obtained with the probe perpendicular to the ramus of the mandible just inferior to the ear. A longitudinal image of the submandibular gland was obtained with the probe parallel and medial to the mandibular body.

**Fig. 1:**
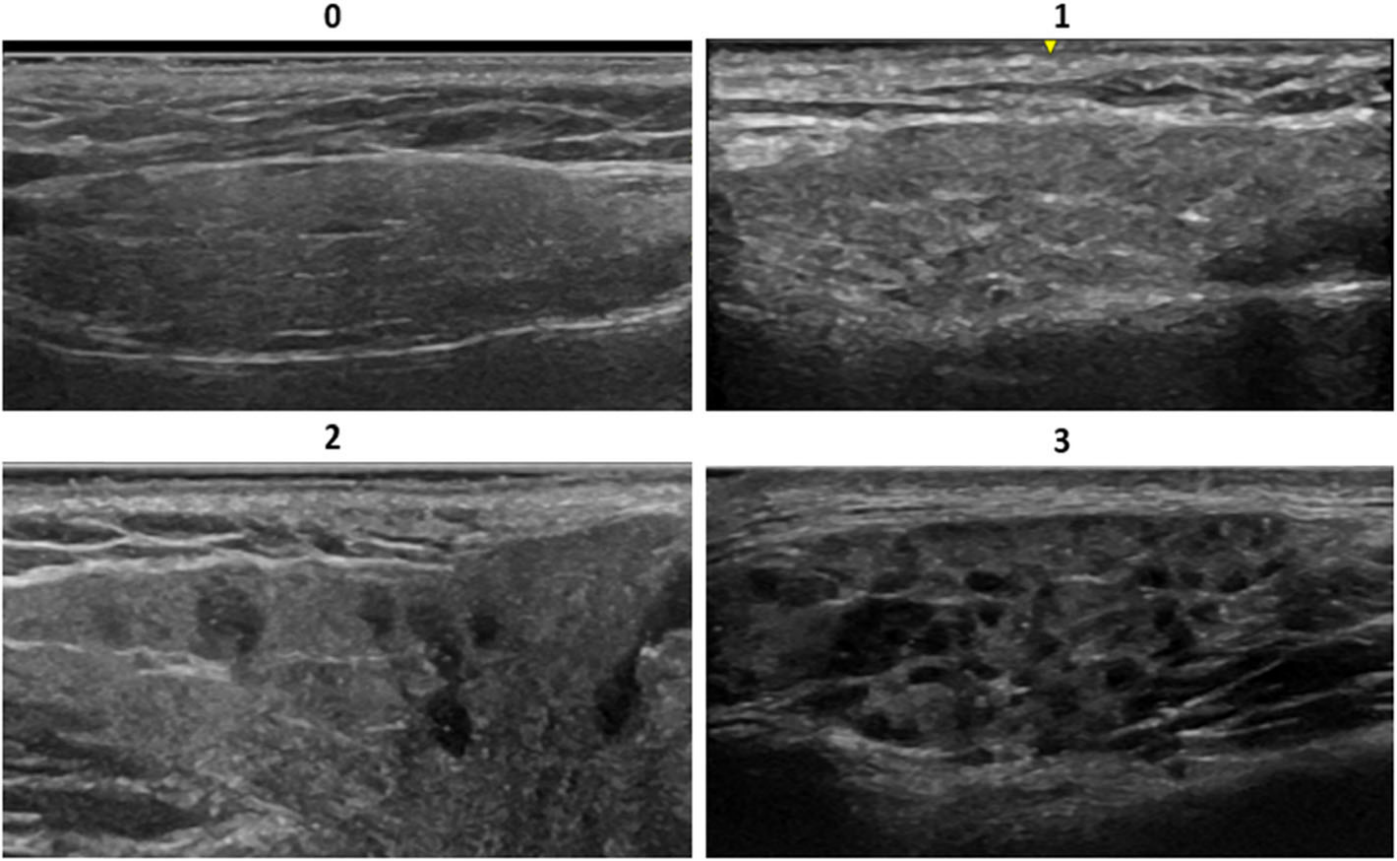
Major salivary gland ultrasonography images of parotid glands illustrating the semi-quantitative scoring system. A score of 0 representing a completely homogeneous parotid gland. A score of 1 representing small, nonspecific punctate areas of heterogeneous gland echogenicity. A score of 2 shows moderate areas of heterogeneous gland echogenicity. A score of 3 shows diffuse heterogeneous gland echogenicity. Scores of 0 and 1 are considered normal. Scores of 2 and 3 are pathologic and represent a pattern that can be seen in Sjögren’s syndrome

**Fig. 2:**
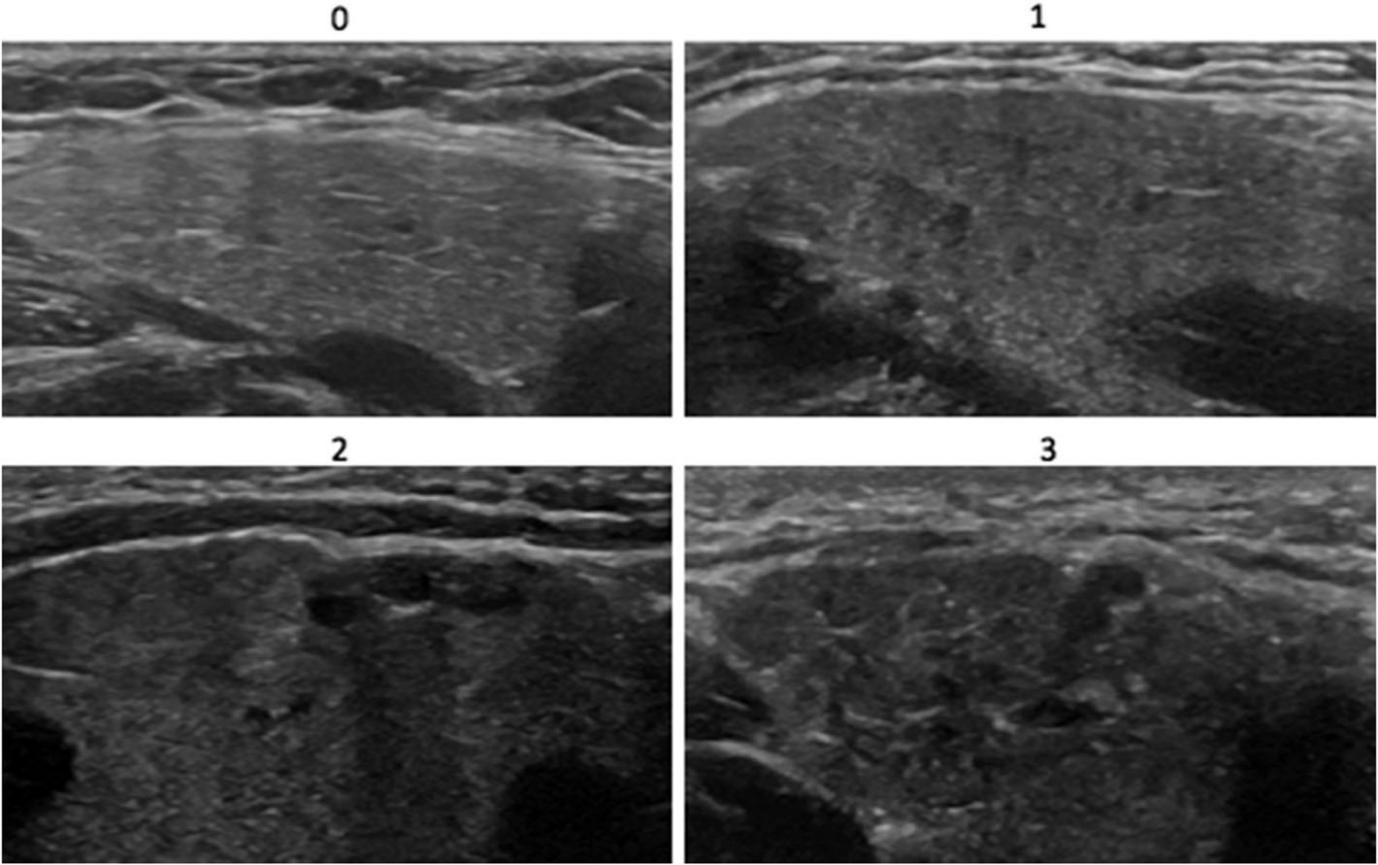
Major salivary gland ultrasonography images of submandibular glands illustrating the semi-quantitative scoring system. A score of 0 representing a completely homogeneous submandibular gland. A score of 1 representing small, nonspecific punctate areas of heterogeneous gland echogenicity. A score of 2 shows moderate areas of heterogeneous gland echogenicity. A score of 3 shows diffuse heterogeneous gland echogenicity. Scores of 0 and 1 are considered normal. Scores of 2 and 3 are pathologic and represent a pattern that can be seen in Sjögren’s syndrome

The authors performed image acquisition and scoring. All authors are Ultrasound School of North American Rheumatologists (USSONAR) trained with each having attended at least one focused salivary gland ultrasound course. Scoring was performed as a group, with a consensus final score established for each patient after discussion among raters. A previously published semi-quantitative scoring system (grades 0–3) that has been used in adults with SS was used to score each image [12]. A score of 0 representing a normal gland with homogenous echogenicity; a score of 1 representing a gland with small non-specific areas of heterogeneous echogenicity; a score of 2 representing a gland with moderate areas of heterogeneous echogenicity (up to 50% of the gland); and a score of 3 representing a gland with large areas of heterogeneous echogenicity (> 50% of the gland). Scores of 0 and 1 were considered negative for Sjögren’s specific findings. Scores of 2 and 3 were considered positive for Sjögren’s specific findings (Figs. 1 & 2). The highest score of any of the six images obtained for each patient as determined by the panel of three reviewers was used as the final score.

Each patient was asked to provide open-ended feedback on exam acceptability. Ultrasound examination and scoring time were recorded for each patient to assess feasibility of the ultrasound protocol in a clinic setting. Time to complete each ultrasound examination was noted by the start and end time as documented on the images collected. Scoring time was calculated as time to complete the review of all images for each subject.

### Statistical analysis

Statistical analysis was performed using the Statistical Package for the Social Sciences (SPSS) Version 26. Independent two-sample t-tests were used to compare means between continuous demographic and disease variables (age, disease duration, and IgG level). Fisher’s exact or Chi-squared tests were used to compare categorical demographic and disease variables (race, patient symptom responses, anti-dsDNA, anti-Smith, anti-RNP, anti-Ro, anti-La). Mann-Whitney U test was used for non-parametric data (SLEDAI, dryness score, fatigue score, pain score). *P*-values < 0.05 were considered statistically significant.

## Results

### Demographic and disease features

A total of 31 patients with childhood-onset SLE participate in our study. Among the 31 patients, 26 (84%) were female, 17 (55%) were Caucasian, 13 were Black (42%), 1 (3%) was Asian, and 1 (3%) was Hispanic. The average disease duration was 5 years (SD 3.5). Median SLEDAI for all patients was 2.0 (IQR 0.0–4.0), suggesting on average patients had mild disease activity. Mean IgG level at the time of diagnosis, prior to any therapeutic intervention, was 1570.0 (SD 734.5) mg/dL. The majority of patients, 21 of 31, (68%) were anti-dsDNA positive. Anti-RNP was also positive in a majority, 22 of 31 (71%) patients. Anti-Smith antibody was seen in 16 (52%) patients. Anti-Ro antibody was present in 15 (48%) patients and anti-La antibody in 5 (16%) patients (Table 1).

**Table 1:** Demographic and disease characteristics in patients with childhood-onset systemic lupus erythematosus

	*N* = 31 (%)^a^
**Demographics**	
Age, years, Mean (SD)	18.5 (2.6)
Female	26 (84)
Caucasian	17 (55)
Black	13 (42)
Asian	1 (3)
Hispanic	1 (3)
**Disease characteristics**	
Disease Duration, years, Mean (SD)	5.0 (3.5)
SLEDAI^b^, Median (IQR)	2.0 (0.0–4.0)
Anti-dsDNA^c^	21 (68)
Anti-Smith^c^	16 (52)
Anti-RNP^c^	22 (71)
Anti-Ro^c^	15 (48)
Anti-La^c^	5 (16)
IgG^d^ mg/dl, Mean (SD)	1570.0 (734.5)

^a^ Number of patients with percentage unless noted; ^b^SLEDAI: Systemic Lupus Erythematosus Disease Activity Index, most recent value; ^c^ Any history of antibody positivity; ^d^IgG level at disease diagnosis

### Medication use

Medication use in the last month was recorded at the time of each study visit. Almost every patient was on hydroxychloroquine, 30 of 31 (97%). A majority of patients were also on mycophenolate, 22 (71%). There was one patient (3%) receiving treatment with azathioprine and one patient (3%) receiving treatment with cyclophosphamide. 13 (42%) patients were receiving steroids, with a mean prednisone dose of 13.8 mg (Table 2).

**Table 2:** Patient-reported measures, current medication use, and ultrasound examination in patients with childhood-onset systemic lupus erythematosus

	*N* = 31 (%)^a^
**Patient-reported measures**	
Dryness Score^b^, Median (IQR)	2.0 (0.0–4.0)
Fatigue Score^b^, Median (IQR)	2.0 (1.0–5.0)
Pain Score^b^, Median (IQR)	1.0 (0.0–3.0)
Needs water to swallow	7 (23)
Episodic cheek swelling	4 (13)
**Medications**	
Hydroxychloroquine^c^	30 (97)
Mycophenolate^c^	22 (71)
Azathioprine^c^	1 (3)
Cyclophosphamide^c^	1 (3)
Rituximab^c^	0 (0)
Prednisone^c^	13 (42)
Prednisone, mg, Mean dose	13.8
**Ultrasound examination**	
US examination time, minutes, Mean	5.0
US scoring time, minutes, Mean	2.0
Abnormal SGUS score^d^	11 (35)

^a^ Number of patients with percentage unless noted; ^b^Dryness, Fatigue, and Pain scores on a scale of 0–10 with 10 being the worst score; ^c^Medication use within the last month; ^d^Abnormal SGUS score is a score of 2 or 3 as defined by Theander & Mandl [12]

### Patient-reported measures

Each patient was asked to complete symptom questionnaires on a scale of 0–10. The median dryness score was 2.0 (IQR 0.0–4.0). The median fatigue score was 2.0 (IQR 1.0–5.0). Median pain score was 1.0 (IQR 0.0–3.0). When asked if water was needed to help swallow food 7 (23%) patients said yes. Additionally, 4 (13%) patients reported a history of episodic glandular swelling (Table 2).

### Ultrasound examination

The average time to complete the ultrasound examination was 5 min. The average time to score all of the ultrasound images was 2 min (Table 2). There were no instances of clinic delays secondary to ultrasound examination. Patients found the examination protocol acceptable, with 100% reporting the exam was quick and painless. Others stated they enjoyed being able to see the structures we were examining and better understood the purpose of the research study. There were only three patients that chose not to participate in the study and they declined because of research burden and prior to learning about ultrasound examination.

### SGUS imaging and disease associations

Pathologic SGUS findings were observed in 11 of 31 (35%) patients (Table 3). Patients with positive SGUS findings (SGUS+) were slightly older than those with negative SGUS findings (SGUS-), 19.6 years vs. 18.0 years, however this was not statistically significant (*p* = 0.11). Additionally, there was no significant difference in disease duration between the groups. The proportion of females in the SGUS+ and SGUS- groups were similar. There were a higher proportion of Black patients with SGUS+ compared to those with SGUS-, 7 out of 11 (64%) vs. 6 out of 20 (30%), but this was not significant (*p* = 0.13). Patients had mildly active disease on average based on SLEDAI scores and there were no differences between SGUS+ and SGUS- patients, 4.0 vs. 2.0 (*p* = 0.90) (Table 3).

**Table 3:** Demographics, disease characteristics, and patient-reported measures of patients with normal vs. abnormal salivary gland ultrasound studies

	Normal US *N* = 20 (%)^a^	Abnormal US *N* = 11 (%)^a^	*p*-value
**Demographics**			
Age, years, Mean (SD)	18.0 (2.5)	19.6 (2.6)	0.11
Female	18 (90)	9 (82)	1.00
Caucasian	13 (65)	4 (36)	0.15
Black/African American	6 (30)	7 (64)	0.13
**Disease characteristics**			
Disease Duration, years, Mean (SD)	4.5 (3.5)	5.9 (3.6)	0.31
SLEDAI^b^, Median (IQR)	2.0 (0.0–4.0)	4.0 (1.0–5.0)	0.74
Anti-dsDNA^c^	15 (75)	6 (55)	0.24
Anti-Smith^c^	10 (50)	6 (55)	1.00
Anti-RNP^c^	13 (65)	9 (82)	0.43
Anti-Ro^c^	6 (30)	9 (82)	**0.01**
Anti-La^c^	1 (5)	4 (36)	**0.04**
IgG^d^ mg/dL, Mean (SD)	1323.4 (559.6)	2017.3 (825.7)	**0.01**
**Patient-reported measures**			
Dryness Score^e^, Median (IQR)	0.5 (0.0–3.0)	3.0 (1.5–5.0)	0.09
Fatigue Score^e^, Median (IQR)	2.5 (0.8–4.0)	2.0 (1.0–5.5)	0.80
Pain Score^e^, Median (IQR)	1.0 (0.0–3.0)	1.0 (0.0–4.0)	0.86
Needs water to swallow	4 (20)	3 (27)	0.68
Episodic cheek swelling	3 (15)	1 (9)	1.00

^a^ Number of patients with percentage unless noted; ^b^ SLEDAI: Systemic Lupus Erythematosus Disease Activity Index, most recent value; ^c^Any history of antibody positivity; ^d^ IgG level at disease diagnosis; ^e^Dryness, Fatigue, and Pain scores on a scale of 0–10 with 10 being the worst score

SGUS+ patients reported higher median dryness scores than SGUS- patients, 3.0 vs. 0.5, although these results were not statistically significant (*p* = 0.08). There were no differences in median fatigue score or pain scores reported on the patient questionnaire between SGUS+ and SGUS- patients (Table 3). Additionally, no associations were seen between the number of patients reporting needing water to swallow and ultrasound abnormalities.. Rates of reported episodic glandular swelling were comparable with 1 of 11 (9%) in SGUS+ vs. 3 of 20 (15%) of SGUS- patients (*p* = 1.00).

There was an association between SGUS+ patients and the presence of anti-Ro antibody. 9 of 11 (82%) of SGUS+ patients had anti-Ro antibody positivity vs. 6 of 20 (30%) of patients in the SGUS- group (*p* = 0.01). A similar association was seen for anti-La antibody. Additionally, mean IgG at the time of disease diagnosis was higher in SGUS+ patients vs. SGUS- patients, 2017.3 mg/dL vs. 1323.4 mg/dL (*p* = 0.01) (Table 3).

There were no significant differences between rates of anti-RNP, anti-Smith, or anti-dsDNA between the two groups (Table 3). Most patients in both groups were on some form of systemic treatment with a majority being on hydroxychloroquine and/or mycophenolate. There were no differences in medication regimen between SGUS+ and SGUS- patients, including the presence or mean dose of steroids.

## Discussion

This is one of the first studies focused on major SGUS in patients with cSLE who do not have a formal diagnosis of SS. We adapted a previously published ultrasound protocol with a simplified scoring system for our cohort of patients [12]. We demonstrated that this protocol was feasible with performance at the bedside during a regularly scheduled clinic visit. With an average exam plus scoring time of 7 min this examination did not significantly delay overall clinic visit time and we did not have any reported negative effects on clinic flow. Although we did not collect quantitative data of ultrasound examination acceptability, patients universally stated that the examination protocol was acceptable. Pathologic changes were identified on major SGUS in 11 of 31 (35%) patients. The rate of SGUS+ was higher than initially suspected, although given the pilot nature of this study the results were hard to predict. Interestingly, our findings were similar to a recent study in adults evaluating SGUS in patients with connective tissue diseases including: SLE, MCTD, and undifferentiated connective tissue disease (UCTD) (with the majority of patients having SLE). This study found pathologic SGUS findings in 27% of patients with connective tissue diseases including SLE [19]. The authors from this study observed that abnormal SGUS seemed to correlate with oral dryness symptoms and abnormal tear production although this was not observed in our patient population. It is important to note that our cross-sectional study design limited performance of additional validation testing including objective measures of oral and ocular dryness or more definitive salivary gland biopsies. However, abnormal SGUS findings in these patients may be suggestive of subclinical SS. It is important to note that these salivary gland ultrasound abnormalities are not necessarily specific and can be seen in other disorders such as infection, sarcoidosis, IgG4-related disease, amyloidosis, and malignancy. The abnormal findings should be taken in the context of the patient’s clinical presentation and should be further evaluated as necessary.

Abnormal SGUS findings were significantly associated with both the presence of anti-Ro and anti-La antibodies in patients with cSLE. These results are consistent with previous SGUS studies in both adults and children [12–14]. Anti-Ro and anti-La antibodies are known risk factors for the development of SS [20, 21]. Rates of anti-Ro antibodies present in cSLE patients range from 23 to 56% and anti-La antibodies range from 7 to 37% [22–24]. However, there are very few reports of secondary SS in cSLE, mostly confined to case series [5–7]. Our imaging findings may represent subclinical disease that could manifest itself with more overt clinical symptoms as time goes on. There is evidence that patients who develop SS display anti-Ro and anti-La autoantibodies many years prior to clinical symptom development [20]. Interestingly, many of our patients reported some degree of subjective oral and/or ocular dryness even though we did not find differences between groups. This is likely secondary to our small number of enrolled patients. It would be important to determine if these subjective symptoms are associated with objective measures of dryness, particularly in those patients with abnormal ultrasound imaging.

Abnormal SGUS findings were also associated with higher levels of IgG. This finding is consistent with previous adult SGUS data [12]. Furthermore, IgG elevation and rheumatoid factor (RF) have been shown to be more prevalent in patients with SLE who were diagnosed with secondary SS than those with SLE alone [25]. Unfortunately, only 3 of our 31 patients had RF obtained during their disease course so we were unable to assess for this association.

There are several limitations to our pilot study including its small sample size and cross-sectional design. Additionally, this study was performed at a tertiary academic medical center and may not be applicable to all clinic settings. Ultrasound imaging assessments were performed on average 5 years into the disease course with all patients on at least one form of systemic medication therapy. Additionally, the ultrasound protocol was a protocol developed for adult patients so the applicability in a pediatric population is not completely clear. Finally, as mentioned previously, imaging findings to date have not been validated with biopsy or additional measures such as salivary flow testing.

## Conclusions

Despite these limitations, SGUS represents a non-invasive, non-ionizing tool to assess salivary glands in patients with childhood-onset rheumatic disease. It may play a role in the screening evaluation of patients with cSLE to assess for salivary gland disease, which could provide more organ-specific targeted therapies. These patients could undergo additional diagnostic work up if SGUS imaging is found to be abnormal. Fortunately, many therapies that are used to treat cSLE are also effective in the treatment of SS. However, if salivary gland disease is identified, these patients should undergo more frequent screening with dental and eye specialists to monitor for signs of oral or ocular damage and to provide symptom-based therapy to improve quality of life.

Larger studies are needed to further characterize SGUS findings in the childhood-onset lupus population. Future validity studies in this patient population comparing ultrasound findings to salivary gland biopsy and objective measures of dryness such as salivary flow testing are needed. Additionally, a study including a control population would be helpful to characterize the healthy salivary gland in the pediatric population. Finally, prospective, longitudinal studies will help to elucidate if US findings are sensitive to change over time and the correlation of SGUS abnormalities with development of overt clinical symptoms with time.

## Data Availability

The datasets used and/or analyzed during the current study are available from the corresponding author on reasonable request.

## Abbreviations

cSLE: Childhood-onset systemic lupus erythematosus
ESSPRI: EULAR Sjögren’s syndrome patient reported index
EULAR: European League Against Rheumatism
IQR: Interquartile range
MCTD: Mixed connective tissue disease
RF: Rheumatoid factor
SD: Standard deviation
SGUS: Salivary gland ultrasound
SGUS+: Positive salivary gland ultrasound
SGUS-: Negative salivary gland ultrasound
SLE: Systemic lupus erythematosus
SLEDAI: Systemic lupus erythematosus disease activity index
SPSS: Statistical package for the social sciences
SS: Sjögren’s syndrome
UCTD: Undifferentiated connective tissue disease
